# Relationship between acaricide resistance and acetylcholinesterase gene polymorphisms in the cattle tick *Rhipicephalus microplus*[Fn FN1]

**DOI:** 10.1051/parasite/2024003

**Published:** 2024-02-04

**Authors:** Raquel Cossio-Bayugar, Francisco Martinez-Ibañez, Hugo Aguilar-Diaz, Estefan Miranda-Miranda

**Affiliations:** 1 Centro Nacional de Investigación Disciplinaria en Salud Animal e Inocuidad, Instituto Nacional de Investigaciones Forestales Agrícolas y Pecuarias INIFAP Boulevard Cuauhnahuac 8534, Jiutepec Morelos CP 625574 México; 2 Departamento de Ectoparásitos y Dípteros. Servicio Nacional de Sanidad, Inocuidad y Calidad Agroalimentaria SADER Boulevard Cuauhnahuac 8534, Jiutepec Morelos CP 625574 México

**Keywords:** RT-PCR, Protein 3D modeling, Pesticide-resistance, Cattle tick

## Abstract

In this study, we aimed to develop a comprehensive methodology for identifying amino acid polymorphisms in acetylcholinesterase transcript 2 (*AChE2*) in acaricide-resistant *Rhipicephalus microplu*s ticks. This included assessing *AChE2* expression levels through qPCR and conducting 3D modeling to evaluate the interaction between acaricides and AChE2 using docking techniques. The study produced significant results, demonstrating that acaricide-resistant *R. microplus* ticks exhibit significantly higher levels of *AChE* expression than susceptible reference ticks. In terms of amino acid sequence, we identified 9 radical amino acid substitutions in AChE2 from acaricide-resistant ticks, when compared to the gene sequence of the susceptible reference strain. To further understand the implications of these substitutions, we utilized 3D acaricide-AChE2 docking modeling to examine the interaction between the acaricide and the AChE2 catalytic site. Our models suggest that these amino acid polymorphisms alter the configuration of the binding pocket, thereby contributing to differences in acaricide interactions and ultimately providing insights into the acaricide-resistance phenomenon in *R. microplus*.

## Abstract (fr)

Notre étude vise à développer une méthodologie complète pour identifier les polymorphismes d’acides aminés dans le transcrit 2 de l’acétylcholinestérase (*AChE2*) chez les tiques *Rhipicephalus microplus* résistantes aux acaricides. Cela comprend l’évaluation des niveaux d’expression d’*AChE2* via qPCR et la réalisation d’une modélisation 3D pour évaluer l’interaction entre les acaricides et l’*AChE2* à l’aide de techniques d’amarrage moléculaire. L’étude a produit des résultats significatifs, démontrant que les tiques *R. microplus* résistantes aux acaricides présentent des niveaux d’expression d’*AChE* significativement plus élevés que les tiques sensibles de référence. En termes de séquence d’acides aminés, nous avons identifié 9 substitutions d’acides aminés dans *AChE2* provenant de tiques résistantes aux acaricides par rapport à la séquence génétique de la souche sensible de référence. Pour mieux comprendre les implications de ces substitutions, nous avons utilisé la modélisation de l’amarrage acaricide-AChE2 pour examiner l’interaction entre l’acaricide et le site catalytique AChE2. Nos modèles suggèrent que ces polymorphismes d’acides aminés modifient la configuration de la poche de liaison, contribuant ainsi aux différences dans les interactions acaricides et fournissant finalement un aperçu du phénomène de résistance aux acaricides chez *R. microplus*.

## Introduction

Acaricide resistance in the cattle tick *Rhipicephalus microplus* (Canestrini, 1887) (Acari: Ixodidae) presents a persistent and costly challenge in bovine exploitations situated in tropical and subtropical regions [13]. Controlling cattle ticks often involves the extensive use of pesticides, leading to concerns such as environmental and food contamination, as well as the emergence of pesticide resistance [8]. The development of pesticide resistance in arthropods is a complex phenomenon influenced by various factors, including behavioral, biochemical, and metabolic defensive mechanisms aimed at mitigating the impact of pesticides on the target organisms [2–5]. Successful control of cattle ticks hinges on timely diagnosis and the selection of the most efficacious acaricide [8].

Previous research has established a connection between pesticide resistance and the activity of xenobiotic-metabolizing enzymes (XMEs) [2, 5, 6]. These enzymes can be found in various metazoan organisms, where they serve as an enzymatic defense mechanism against the potentially toxic effects of natural xenobiotic compounds [26]. Arthropods, in particular, possess an efficient assortment of XMEs, including cytochrome P450 (CYP), carboxylesterases (CE) [26], and other XMEs that facilitate the conversion of exogenous chemicals into hydrophilic derivatives [26].

Acetylcholinesterase (AChE) is closely related to carboxylesterases, which are XMEs [16]. AChE is present in a large range of organisms, including arthropods, where it regulates levels of acetylcholine in muscular tissues [6]. The active site of AChE can be phosphorylated by organophosphorus pesticides (OPs), such as the acaricide diazinon [21], leading to the inactivation of AChE as a desired toxic effect in arthropods [34]. In some cases, arthropods develop a form of pesticide resistance involving a mutated version of AChE that can resist phosphorylation at the active site by OP pesticides. This resistance mechanism has been observed in *Musca domestica* (Diptera: Muscidae) [20], *Culex pipiens* (Diptera: Culicidae) [33], *Anopheles albimanus* (Diptera: Culicidae) [12], and *Drosophila melanogaster* (Diptera: Drosophilidae) [4]. A different category of acaricide-resistant enzymes known as cholinesterases and carboxylesterases (CEs) are involved in sequestering OP pesticides, and increasing the expression of acetylcholinesterase (AChE) [2, 12, 13]. These closely related enzymes likely contribute to enhanced detoxification through ester hydrolyzing activity, as evidenced by their increased expression [13–15, 18, 19]. Additionally, both enzymes share an affinity for various synthetic substrates [24]. In cattle ticks, at least three *AChE* transcripts have been identified: *BmAChE1* [3], *BmAChE2* [16], and *BmAChE3* [31]. Notably, increased transcript expression of *BmAChE2* has been observed in field-isolated OP acaricide-resistant ticks from Mexico [11] and Brazil [5], which suggests a potential role in acaricide resistance.

The objective of this study was to establish a methodology for assessing the expression levels of XMEs in acaricide-resistant *R. microplus* ticks, recognizing the significance of XMEs in acaricide resistance.

## Materials and methods

### Ethics

The study was conducted according to the guidelines of the Declaration of Helsinki and supervised by the Experimental Animals Handling Ethics Committee of the Instituto Nacional de Investigaciones Forestales Agricolas y Pecuarias. The study design and experimental protocols were performed according to Mexican standard NOM-062 ZOO-1999 for animal care and use, and the technical specifications for the production, care and use of laboratory animals used can be found at https://fmvz.unam.mx/fmvz/principal/archivos/062ZOO.PDF.

### Ticks

For baseline levels of cholinesterase and carboxylesterase gene expression, an acaricide-susceptible reference strain (SUS) that has been maintained without exposure to any acaricides since 2008 was utilized. In addition, a multiple resistance reference strain that has been cultured for multiple generations has served as the resistance reference in bioassays conducted as part of the Mexican Federal Government’s acaricide resistance monitoring program [25]. The field isolates of cattle ticks were collected from cattle ranches located in the southeastern Mexican state of Tabasco as part of the compulsory inspection of cattle for the acaricide resistance monitoring program. All tick specimens were subsequently maintained at the Department of Ectoparasites and Diptera of the National Service for Agro-Alimentary Public Health, Safety and Quality (SENASICA) under the supervision of the Secretariat of Agriculture and Rural Development (SADER) in Mexico. The susceptible and acaricide-resistant reference strains, as well as the tick field isolates utilized in this study, were obtained by infesting cattle with 2 × 10^4^ 10–15-day-old larvae. Engorged females were collected 21 days after infestation and placed in Petri dishes, with each strain represented by groups of ten ticks, for subsequent oviposition. The Petri dishes were then incubated at a temperature of 28 °C and 80% relative humidity until complete oviposition, following established protocols [7]. The tick egg masses were subsequently collected, weighed, and divided into 200 mg vials. These vials were kept at a temperature of 28 °C and 80% relative humidity until eclosion. Meanwhile, the 10-day-old larvae were frozen at −80 °C and stored for future use.

### Larval package test bioassay

The toxicological profiles of the reference strains and isolates were assessed for their resistance to organophosphate acaricides using bioassays at the Laboratory of the Ectoparasites and Dipteran Department of the National Animal Health Verification Services Center (SENASICA-SADER) [30]. The larval test employed in this study involved exposing tick larvae to filter papers impregnated with acaricides at predetermined concentrations capable of causing 99% mortality in susceptible tick populations (LD99) after 24 hr [25]. Four replicates were used for each reference tick strain and isolate tested, with trichloroethylene-diluted acaricides administered at the following concentrations: chlorpyrifos 0.2%, coumaphos 0.2%, and diazinon 0.08%. To impregnate the filter papers, a 63 cm^2^ piece of Whatman 1 filter paper was treated with one milliliter of each acaricide dilution. Once the trichloroethylene evaporated, the treated filter papers were sealed on three sides with clips, and one hundred 10-day-old larvae were introduced through the open side, which was then sealed with another clip. After incubating for 24 hr at 28 °C and 92% relative humidity, live and dead larvae were counted [25]. The mortality rate for each tick group under each acaricide concentration was recorded as data, as shown in Table 1.

**Table 1:** Acaricide bioassay data of different tick field isolates. Larvae from different strains and isolates were bioassayed by a larval package test under standard concentrations of the acaricides diazinon coumaphos and chlorpyrifos. The data are presented as the mortality rate (%) under a standard acaricide concentration.

	Organophosphate		
Tick sample	Chlorpyrifos	Coumaphos	Diazinon
Susceptible	100	100	100
Resistant	0	0	0
Isolate C1	62.93	100	0
Isolate C2	42.1	100	0

### Relative quantification of cholinesterase expression

Each sample of *R. microplus* ticks was frozen at −80 °C and subsequently finely ground using a ceramic mortar. Total RNA was isolated using an RNAqueous^®^-4PCR Kit (Ambion, Austin, TX, USA), following the manufacturer’s instructions. The isolated RNA was then transcribed into cDNA using random decamer primers according to the instructions of a High-capacity cDNA Reverse Transcription Kit, a commercially available kit from Applied Biosystems.

TaqMan^®^ probes for *acetylcholinesterase transcript 2* (*AChE2)* were synthesized, and real-time PCR was conducted following the methods outlined in [9, 11]. Real-time PCR was performed with a fluorogenic 5’ nuclease assay (TaqMan^®^ system) on an ABI Prism 7300 Sequence Detector (Applied Biosystems, Foster City, CA, USA). The gene-specific PCR primers for *AChE2* and the TaqMan^®^ probe labeled with 6-carboxyfluorescein (FAM)/MGB were as follows: AChE2For 5′-GGCACTGAAATGGATCCAGGAA-3′, AChE2Rev 5′-CGTGACTTCACCAGGGTTACC-3′, and the AChE2 TaqMan^®^ probe 5′-CCAAATGCAGCAATGTT-3′. The *R. microplus* eukaryotic endogenous control, 18S rRNA (VIC^®^/MGB^®^; Applied Biosystems), was used as previously reported [9, 11]. The TaqMan^®^ probes were designed using the reported DNA sequences for *BmAChE2* (GenBank accessions AJ278345.1, AJ278344.1, AJ278343.1, AJ278342.1, OR378375.1, and OR378376.1). Real-time PCR analysis was conducted twice in independent experiments, each with four replicates.

The *AChE2* gene expression of each strain and field isolate was measured using 7300 SDS Software v1.2.2 (Applied Biosystems). The expression levels of CYP, CE, and AChE2 in the different samples were quantified using the ΔΔ Ct method, with 18S ribosomal RNA expression levels serving as the internal control for normalization. The susceptible strain was considered to have a baseline level of *AChE2* expression and was assigned a relative value of 1 expression unit (1 REU), following the instructions provided in the ABI Prism 7300 Sequence Detector real-time thermal cycler manufacturer’s manual (Applied Biosystems) available at https://assets.thermofisher.com/TFS-Assets/LSG/manuals/cms_042380.pdf.

### Statistical analysis

The means of the relative *AChE2* gene expression in REU were statistically analyzed using an unpaired Student’s *t*-test conducted with GraphPad Software (GraphPad Software, Inc., La Jolla, CA, USA), which is available online at https://www.graphpad.com/quickcalcs/ttest1.cfm.

### *Rhipicephalus microplus* acetylcholinesterase amino acid sequences

All sequences analyzed in this study were obtained from the NCBI and originated from diverse sources and geographical locations. The AChE2 amino acid sequences derived from the ticks used in the toxicological bioassay were previously submitted to GenBank with the following identifiers: susceptible for AAC18857.1, isolate C1 for OR378375.1, and isolate C2 for OR378376.1.

### Phylogenetic analysis of acetylcholinesterase 2

The amino acid sequences KT215342.1, CAB93509.1, OR378375.1, OR378376.1, and AAC18857.1 were retrieved from the GenBank database (www.ncbi.nlm.nih.gov) for phylogenetic analysis. The Clustal Omega algorithm was employed for sequence alignment, and the analysis was conducted online at https://www.ebi.ac.uk/Tools/msa/clustalo/ [23].

### Acetylcholinesterase 3D modeling

The UniProt amino acid sequences Q9NFK3, A0A0M4JB02, and O61864, along with their respective Alphafold 3D models in PDB files, were downloaded from https://www.uniprot.org [32] and https://alphafold.com [17]. For proteins without available 3D models, the corresponding Fasta amino acid sequences were submitted to https://robetta.bakerlab.org [1]. The complementary GenBank amino acid sequences KT215342.1, CAB93509.1, OR378375.1, OR378376.1, and AAC18857.1 were obtained from www.ncbi.nlm.nih.gov for phylogenetic analysis and construction of the unavailable 3D models. A 3D model of the oxidized acaricide ligand diazoxon was downloaded from https://pubchem.ncbi.nlm.nih.gov. Protein 3D modeling was performed using the Mol* online algorithm [29] available at https://molstar.org. Ligand docking, identification of the ligand-binding site, and 3D modeling of the ligand-binding site were performed using the CB-Dock online algorithm [22] at http://clab.labshare.cn/cb-dock/php/index.php.

## Results

### Acaricide bioassays

The mortality rate of tick larvae exposed to standard concentrations of the acaricides chlorpyrifos, coumaphos, and diazinon was determined to be 100% compared to that of the susceptible reference tick strain. As expected, the resistant reference strain displayed complete resistance with 0% mortality when exposed to all the chemical formulations. Isolates C1 and C2 demonstrated 0% mortality from the acaricide diazinon, although isolate C1 only exhibited partial resistance against chlorpyrifos. However, both isolates exhibited 100% mortality when exposed to coumaphos. Additional details and data can be found in Table 1.

### Acetylcholinesterase 2 expression

The difference in *AChE2* expression between the means of each isolate and resistant strain of ticks and the susceptible strain was statistically significant, which was considered the baseline expression level of 1 REU. Isolates C1 and C2 exhibited significant overexpression levels of *AChE2*, ranging from 8.52 (*t* = 10.875, df = 6) for isolate C1 to 79.59 (*t* = 10.477, df = 6) for isolate C2. The *AChE2* expression level in the resistant reference strain was determined to be 13.07 REU (*t* = 10.875, df = 6). Additional details and data can be found in Table 2.

**Table 2:** Relative quantification of *acetylcholinesterase transcript 2* (*AChE2*) expression levels in ticks. Tick larvae from different strains and isolates subjected to RNA extraction and qPCR, and the results were subsequently converted to REU and compared against those of the susceptible strain, which was considered 1 REU.

Tick sample	AChE2 REU
Susceptible	1 ± 0.3
Resistant	13.07 ± 3.49**
Isolate C1	8.52 ± 1.35*
Isolate C2	79.59 ± 15*

*p < 0.0001; **p < 0.0005.

### Phylogenetic analysis of acetylcholinesterase 2

**Figure 1:**
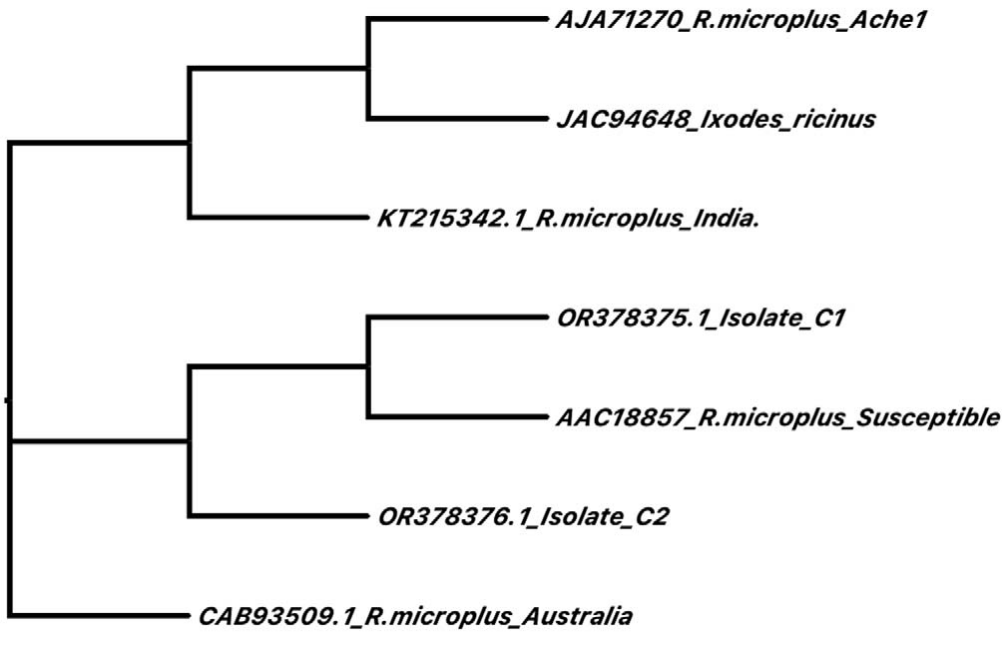
Phylogenetic analysis of AChE2 amino acid sequences from *R. microplus* reported in GenBank. The protein amino acid sequences were submitted online to multiple sequence alignment via the Clustal Omega algorithm at https://www.ebi.ac.uk/Tools/msa/clustalo/, and the results were analyzed via phylogenetic comparison via the neighbor-joining method without distance corrections.

The Clustal Omega multiple sequence alignment algorithm was utilized to construct a phylogenetic tree using the Neighbor-joining method without distance corrections. The resulting phylogeny revealed distinct regions in which *AChE2* sequences are sorted, notably, that the susceptible strain, as well as isolates C1 and C2 from Mexico, formed a distinct clade. Additionally, orthologous *AChE2* sequences obtained from ticks in Australia and India were observed to cluster separately in their own distinctive clades. For a visual representation, refer to Figure 1.

### 3D modeling and AChE2-acaricide docking

**Figure 2:**
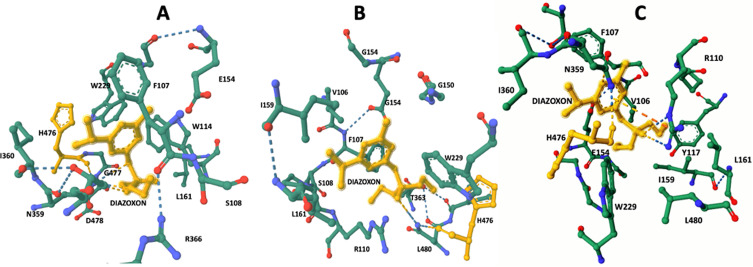
AChE2 ligand-binding site interaction with the oxidized acaricide diazoxon. Diazinon is metabolically oxidized by tick enzymes, enhancing its toxic effect on AChE2 which interacts with the acaricide at the ligand-binding site via hydrogen bonds. (A) Diazoxon highlighted in yellow, which interacts by means of hydrogen bonds with amino acids D478 and G477 in close proximity to H476, an important component of the AChE2 catalytic triad. (B) Isolate C1 AChE2 interacts with L480 and H476. (C) Isolate C2 AChE2 interacts directly with diazoxon via ionic bonds; additionally, the acaricide also interacts via hydrogen bonds with R110 and N359.

The docking 3D model of *AChE2* sequences revealed significant differences at the ligand-binding site when the metabolically oxidized form of diazinon, known as diazoxon, was used to model amino acid level interactions with the acaricide via the CB-Dock algorithm (Table 3 and Fig. 2). In the case of susceptible *AChE2*, diazoxon interacted with the ligand-binding site through hydrogen bonds with amino acids D478 and G477, which are located near H476, an essential component of the *AChE2* catalytic triad. On the other hand, Isolate C1 AChE2 interacted with amino acids L480 and H476, and Isolate C2 *AChE2* uniquely interacted with diazoxon directly via an ionic bond with amino acid H476. Additionally, the acaricide interacted via hydrogen bonds with R110 and N359. These findings indicate that all three analyzed *AChE2* polymorphisms include a distinct set of amino acids that interact with the acaricide at the ligand-binding site.

**Table 3:** Amino acids at the binding pocket and type of bonds involved. Different polymorphisms of *AChE2* result in different amino acid compositions in the binding pocket, which in turn results indifferent types of bonds with diazoxon ligand.

Strain/Isolate	Binding pocket	Hydrogen bonds	Hydrophobic bonds	Ionic bonds
Susceptible	F107, S108, W114, G150, E154 L161, W229, N359, I360,T363, R366, H476, D478, G477	E154-G150	W114-L-161	E154-Diazoxon
		R366-F107	W114-W229	
		N359-T363	I159-L161	
		T363-I360	F107-T363	
		H476-N359	W114-Diazoxon	
		G150-E154	I360-Diazoxon	
		N-Diazoxon	W229-Diazoxon	
			T363-Diazoxon	
			F107-Diazoxon	
C1	V106, F107, S108, P109, R110, G149, G150, E154, G155, I159, L161, W229, N359, I360, T363 H476, L480	H476-E356	W229-Diazoxon	G154-Diazoxon
		H476-N359	I360-Diazoxon	R110-Diazoxon
		T363-I360	T363-Diazoxon	
		N359-T363	P109-Diazoxon	
		T364-I360	L161-Diazoxon	
		Y362-S358	I159-Diazoxon	
		L361-G357		
		Y355-S368		
		G357-L361		
		S358-Y362		
		I360-T364		
		N359-Diazoxon		
C2	V106, F107, S108, P109, R110, Y117, G149, G150, E154, G155, I159, L161, W229, N359, I360, T363, H476, G477, L480	H476-N359	F107-T363	H476-Diazoxon
		N359-T363	I159-L161	N359-Diazoxon
		I360-T363	F107-Diazoxon	R110-Diazoxon
		Y362-S358	W229-Diazoxon	
		G357-L361	V106-Diazoxon	
		H476-E356	L161-Diazoxon	
		F472-S368	I159-Diazoxon	
		N355-S358	R110-Diazoxon	
		D359-Diazoxon	T363-Diazoxon	
		R110-Diazoxon		

## Discussion

Pesticide resistance in arthropods is often determined by a genetic mechanism that may involve the collaboration of one or more genes to achieve resistance. The phenotypes resulting from these genes could include changes in the pesticide target site [6] or an increase in enzymatic detoxification [28]. To investigate this further, our study focused on analyzing the AChE2 amino acid sequences and expression levels in different tick isolates with varying resistance levels. The results of our study revealed variations in both the amino acid sequences and expression patterns of *AChE2* between reference strains and isolates with different toxicology profiles. These findings suggested that these variations are responsible for the varying levels of acaricide resistance found in ticks. The sequence polymorphisms and expression profile of *AChE2* in acaricide-resistant ticks are consistent with the idea of an altered active site of the target protein, as well as the enzymatic detoxification hypotheses. It is possible that the amino acid polymorphisms in *R. microplus AChE2* may bind to, sequester, and enzymatically neutralize acaricides [2, 5, 14, 15, 17–19]. Previous studies have shown increased expression of *AChE2* in OP acaricide-resistant reference strains [11] and field isolates [5]. However, isolate C2 exhibited an unprecedented level of expression, as shown in Table 2. The overexpression of *AChE2* aligns with the hypothesis of OP acaricide sequestration, where excessive enzyme expression ensures sufficient active enzymes for the regulation of acetylcholine nerve impulses even after exposure to OP acaricides, preventing paralysis and death in resistant ticks. This specific transcript is also expressed primarily in the synganglion, the target organ of diazinon [3]. Other studies have also reported an increase in *AChE2* in a Brazilian field isolate resistant to diazinon [5]. These findings suggest that the transcript level of this gene could serve as a discriminative marker between susceptible and OP-resistant ticks. Furthermore, the observation of an 80-fold increase in *AChE* expression in tick isolate C2 suggested that this specific transcript may be associated with an enzyme involved in the sequestration of organophosphates rather than metabolizing them. It has previously been reported that field isolates exhibiting resistance to pyrethroids also display susceptibility to organophosphates (OPs), such as coumaphos and chlorpyrifos, with some isolates demonstrating moderate resistance to diazinon with *AChE2* subexpression [9]. In this particular scenario, the main selection pressure was toward pyrethroid molecules, and it appears that *AChE2* overexpression is not the predominant defense mechanism responsible for the observed moderate diazinon resistance. To gain a deeper understanding, further studies focusing on field isolates with different acaricide levels of selection pressure are needed. These studies should aim to determine whether there are alternative first-response mechanisms apart from AChE2 or if a multifactorial response is involved, indicating that *AChE2* overexpression is not the sole mechanism or could develop later under increased selection pressure. To further understand this phenomenon, it would be beneficial to conduct additional studies on isolates exhibiting similar expression levels. These studies could help determine whether these high levels of expression are achieved through gene duplication, similar to what has been observed in *C. quinquefasciatus* mosquitoes [27]. However, based on our experience, this type of acaricide resistance in *R. microplus* is believed to be influenced by various factors. An increase in AChE may be one contributing factor among others within a complex scenario observed under field conditions [10].

Diazoxon is a metabolically oxidized form of diazinon that undergoes biotransformation primarily at the nervous tissue level. This biotransformation enhances the toxic effect of the acaricide on AChE2, as reported by Lazarević-Pašti *et al*. [21]. Therefore, in our study, we used diazoxon for ligand docking assessment via 3D modeling analysis to evaluate how AChE2 amino acid polymorphisms affect ligand binding sites. Interestingly, the amount of interacting amino acids showed notable changes in all cases, except for H476, as depicted in Figure 2. This particular amino acid, along with S230 and E356, forms the catalytic triad within the catalytic site of all acetylcholinesterases enzymes. This triad is crucial for the hydrolysis of acetylcholine, which regulates the tick’s nervous impulse muscle, as described by Hernandez *et al.* [16]. Our findings suggest a correlation between acaricide resistance and specific amino acid substitutions at the AChE2 catalytic site. Different amino acid polymorphisms result in varying levels of interaction between the acaricide and the catalytic triad. Through our analysis, we observed several amino acid substitutions (as shown in Table 3) in the AChE2 amino acid sequence of ticks with different levels of acaricide resistance. These substitutions inevitably impact the configuration of amino acids at the catalytic site of the enzyme, as depicted in Figure 2. Organophosphorus acaricides exert their toxic effects on ticks through the irreversible phosphorylation of S230 at the catalytic triad. However, prior to this phosphorylation, E356 and H476 play crucial roles by coordinating a nucleophilic attack on the phosphate within the acaricide, as described by Hernandez *et al*. [16]. Based on our data, we propose a scenario in which amino acid reconfiguration at the catalytic site leads to distinct ligand binding interactions specifically around H476, when the identified polymorphisms are modeled through docking analysis. This altered amino acid configuration results in a change in the level of interaction within the catalytic triad in the presence of the acaricide, ultimately rendering the AChE2 polymorphisms resistant to phosphorylation. This phenomenon is illustrated in Figure 2.

Our study revealed a significant level of polymorphism in the *AChE2* gene of *R. microplus* ticks, particularly in acaricide-resistant individuals. Notably, we consistently identified three amino acid substitution polymorphisms (D299, I398, and F546) that contribute to a reconfiguration of the active site. This reconfiguration alters the level of interaction between the acaricide and the catalytic triad of amino acids in ticks, which exhibit high levels of acaricide resistance. These findings hold potential implications for the development of a molecular test for acaricide resistance using *AChE2* qPCR or SNTP analysis.

## Funding

This research was funded by the INIFAP project (SIGI number 1237511601).

## Conflict of interest

The authors declare that the research was conducted in the absence of any commercial or financial relationships that could be construed as a potential conflict of interest.
